# Pharmacotherapy in Pediatric Neurogenic Bladder Intravesical Botulinum Toxin Type A

**DOI:** 10.5402/2012/763159

**Published:** 2012-06-07

**Authors:** Cristian Sager, Carol Burek, Victor Durán, Juan Pablo Corbetta, Santiago Weller, Bortagaray Juan, Juan Carlos López

**Affiliations:** Urology Department, Hospital de Pediatría S.A.M.I.C. “Prof. Dr. Juan P. Garrahan”, Combate de los Pozos No. 1881 (CPA: C 1245 AAM), Buenos Aires, Argentina

## Abstract

When the neurogenic bladder is refractory to anticholinergics, botulinum toxin type A is used as an alternative. The neurotoxin type A reduces bladder pressure and increases its capacity and wall compliance. Additionally, it contributes to improving urinary continence and quality of life. This novel therapy is ambulatory with a low incidence of adverse effects. Due to its transitory effect, it is necessary to repeat the injections in order to sustain its therapeutic effect. In these review article we talk about Mechanism of Action, Indications, effects, administration and presentations of the Botulinum Neurotoxin Type A in pediatric patients. Also, we make references to controversial issues surrounding its use. A bibliographic search was done selecting articles and revisions from Pubmed. The key words used were botulinum toxin A, neurogenic bladder, and children. The search was limited to patients younger than 18 years of age and reports written in English in the past ten years.

## 1. Introduction

This is a review of published work. In most cases, the neurogenic bladder in children is sequela of spinal dysraphism. Less frequent causes include sacral anomalies and traumatic and tumoral lesions of the nervous system.

Children with open dysraphism can present different patterns of behavior in the lower urinary tract. In the case of bladders with high risk of upper urinary tract deterioration, it is essential to confirm the presence of overactivity of the detrusor muscle, with reduction in bladder capacity and urinary incontinence; in other cases, reduced compliance is involved. Detrusor-sphincter dyssynergia (DSD) can be present in 50% of the cases. If it is not treated, it represents and important risk factor, since it will naturally lead to the development of ureterohydronephrosis in over 70% of cases, and vesicoureteral reflux in 80% of cases, with renal parenchyma deterioration [1, 2].

To achieve a urinary reservoir of adequate capacity, low storage and voiding pressure becomes the main goal. In this way, it is possible to preserve the upper urinary tract undamaged. Then, the focus of attention lies on achieving urinary continence and improving the quality of life of these patients.

The classical treatment for the neurogenic bladder in children consists of clean intermittent catheterization (CIC) programs and administration of anticholinergic drugs. Approximately, 90% of patients respond well to this type of therapeutic scheme [3, 4]. However, a percentage of patients, which should not be underestimated, are refractory to this program or develop intolerance to the anticholinergic drugs and thus require lower urinary tract reconstructions, such as bladder augmentation, in order to achieve continent urinary reservoirs with adequate capacity and low pressure.

Two decades ago, intravesical botulinum toxin type A (BTX-A) was first used as a therapeutic alternative. In 1990 it was used in adults with spinal cord injury and DSD [5]. Later on, it was expanded to patients with overactivity of the neurogenic detrusor muscle [6].

BTX-A was not used in children with neurogenic bladders until recently, but today its efficacy can be confirmed, especially in the case of children with overactive detrusor. Thus, this modality has become second-line treatment for patients who are refractory to anticholinergic drugs [7].

## 2. Pathophysiology of Detrusor Overactivity

In healthy individuals, unmyelinated bladder afferent C-fibers in the suburothelium respond at high thresholds to mechanical stimuli, such as volume distension. On the other hand, these fibers of nociceptive nature respond only to irritating stimuli, like chemical, pH, or temperature changes [8].

When there is imbalance and interruption of the afferent and efferent pathways between nervous system and bladder, the micturition reflex and the pathways involved are reorganized, but aberrations in different neural routes occur. In this way, the afferent C-fibers can exhibit a low threshold to mechanical stimuli, like bladder distension at low volumes, and they can excite the spinal cord, with the subsequent parasympathetic release of acetylcholine and erratic contractions of the detrusor muscle. This excitatory disorder is the pathophysiologic basis of neurogenic detrusor overactivity.

The central mechanisms may also be involved in bladder pathology. There is evidence about the effects of the brain on the bladder, especially in the social stress. The psychosocial stressors can produce voiding dysfunctions and bladder pathology via corticotrophin [9].

The disruption of the spinobulbospinal pathway may also result in dyscoordination of the bladder-sphincter complex, resulting in a nonrelaxing external urethral sphincter during detrusor contraction: detrusor-sphincter dyssynergia [10].

## 3. Botulinum Neurotoxin Type A—Mechanism of Action

Botulinum toxin is made by the anaerobic, gram-positive bacteria* Clostridium botulinum* and released as a 150-kDa polypeptide chain. The toxin has a zinc protease that cleaves fusion proteins and it becomes activated by selective proteolytic cleavage to yield the heavy (100 kDa) and light (50 kDa) chains that are linked by a single disulphide bond. The heavy chain is responsible for binding to the nerve terminal at the neuromuscular junction. The light chain, which is internalized by endocytosis, actively cleaves a specific site on the protein complex SNAP-25 (Synaptosomal-associated protein 25), responsible for docking and releasing vesicles containing neurotransmitters into the neuromuscular junction. Acetylcholine is one neurotransmitter which naturally generates muscular contraction [11]. By cleaving protein receptors within the nerve terminals, botulinum toxin prevents the normal vesicular transport and release of acetylcholine from the motor nerve terminals into the neuromuscular junction. As a consequence, muscular contractility is eliminated causing quick relaxation and prolonged local effect. Thus, a chemical muscular denervation is achieved.

Neurotoxin A also expands its inhibitory effects to several proteins collectively called SNAREs (soluble *N*-ethylmaleimide-sensitive factor attachment protein receptors), on which depend the release of neurotransmitters and neuropeptides involved in sensory neurotransmission in response to mechanical stimuli, such as adenosine triphosphate (ATP), substance P, calcitonin gene-related peptide (CGRP), and glutamate. A reduction in the levels of nerve growth factor (NGF) in the bladder wall has also been reported. Hence, it is likely that BTX-A also modulates intrinsic bladder reflexes through a multimodal effect on sensory pathways [12].

Additionally, the actions of myofibroblasts located on the suburothelium and urothelium have been studied as elements involved in pathways of bladder mechanosensation. It has been concluded that BTX-A may exert a multimodal effect on those pathways via multiple inhibition of the vesicular release of neurotransmitters and neuropeptides [12]. These mechanisms could explain, in part, the effects of the toxin on irritative bladder symptoms, such as voiding urgency.

## 4. History—Antecedents

In 1990 botulinum toxin type A was used for the treatment of detrusor-sphincter dyssynergia in patients with spinal cord injury with successful results [5]. Years later, intradetrusor BTX-A injections were used in adult patients with neurogenic overactivity, with encouraging results [6].

In 2002 the use of botulinum toxin was reported for the first time in 17 children with detrusor overactivity of neurogenic origin. The authors concluded that BTX-A injection in patients with detrusor hyperreflexia increased bladder capacity, reduced pressure, and improved compliance, with good tolerance. They asserted that the use of this toxin could become an alternative therapy to anticholinergic drugs [7]. Two years later, similar results in urodynamic parameters were reported by Riccabona et al., but in this latter study the impact of injections on the urinary incontinence score was also highlighted [13]. 

## 5. Indications for Pediatric Patients

These indications are as follows.

- Neurogenic detrusor overactivity.
- Idiopathic detrusor overactivity.
- Bladder overactivity syndrome.
- Detrusor-sphincter dyssynergia.
- Reduced bladder compliance (still under revision).

## 6. Effects

### 6.1. Clinical Parameters

In patients with neurogenic bladder that are under a clean intermittent catheterization program, an important, although variable, reduction in incontinence episodes has been reported. Between 65% and 85% improvement in urinary continence score has been reported after intradetrusor BTX-A injection [13–16]. Likewise, an increase in urinary volumes recorded in voiding diaries was also observed [17].

### 6.2. Urodynamic Variables

All the studies analyzed show a significant reduction in Pdetmax (maximum detrusor pressure during bladder filling) of 33% to 55%, and in several studies it reached values lower than 40 cm H_2_O. The maximum cystometric capacity increased between 35% and 80%, with basal values between 110 and 200 mL. In addition, detrusor compliance exhibited an increase between 58% and 180%. Several studies reported compliance values greater than 20 mL/cm H_2_O [13–16]. Despite the promising results so far mentioned here, there is still a need for controlled studies with a bigger sample size (level of evidence: 3) [18].

## 7. Administration of Botulinum Toxin Type A in the Detrusor Muscle

In general, the dose used is 5 to 10 U/kg of body weight; maximum dose: 300 U; dilution: 10 U/mL. These values were extrapolated from other uses of this toxin in neuropediatrics. Caution should be exercised regarding the total dose administered to children, especially when botulinum toxin is also used to treat spasticity. In the early series, the toxin was injected at 30 to 45 intradetrusor sites, following an imaginary parallel line system all around the endovesical surface, sparing the trigone, since its inclusion could increase the possibility of generating vesicoureteral reflux. In recent studies, fewer sites were injected (around 10) and they included the trigone, with acceptable therapeutic results, like 30–45 sites [19].

The depth of the injection can be suburothelial or intramuscular. Both techniques can increase cystometric capacity. The technique has not been standardized. Rigid and flexible cystoscopes have been used. For the flexible cystoscope, an ultrafine 4 mm needle (Dasgupta technique) can be used, which is less invasive. At present, there are no comparative studies published that analyze this aspect of injection. There is, however, evidence of an intention to simplify the injection technique, for instance with the use of different devices, such as endoinjector needle systems, with curved needles that can reach difficult endovesical sites, like lateral walls or bladder roof and trabecular region [20, 21].

Moreover, efforts to avoid the use of general anesthesia during the injection procedure have been made. Thus, the introduction of botulinum toxin into the detrusor by means of 3 electrokinetic phenomena: (a) iontophoresis: method of transmitting charged drugs though the tissue using an electrical current; (b) electroosmosis: introduction of nonionized molecules to the hydratation shells of ionized particles; (c) electroporation: the permeability of the tissue is increased [22]. This is an easy and safe method of incorporating the neurotoxin into the bladder, especially in the case of children with neurogenic bladder due to myelomeningocele [22] The introduction of the toxin under general anesthesia would be reserved for adolescent patients, patients who are anxious, patients with nonneurogenic or idiopathic bladder dysfunction, or those with risk of autonomic dysreflexia.

## 8. Botulinum Toxin Type A Presentations

In 2009 the FDA (Food and Drug Administration) issued a warning statement describing the different botulinum toxins types currently in use in the market stating that: “The revised labels also emphasize that the different botulinum toxin products are not interchangeable, because the units used to measure the products are different. To help reduce the potential for dosing errors, the botulinum toxin products have changed their established drug names (often referred to as the drug's “generic” name). Neither the brand names nor the formulations of the products have changed. Therefore, the new names of active principles and brands are the following:

- OnabotulinumtoxinA (marketed as Botox/Botox Cosmetic).
- AbobotulinumtoxinA (marketed as Dysport).
- RimabotulinumtoxinB (marketed as Myobloc).
- IncobotulinumtoxinA (marketed as Xeomin).”

Despite the increasing evidence for the use of BTX-A in the urinary tract and its proven efficacy, this therapeutic alternative is currently not licensed for the treatment of lower urinary tract and pelvic floor disorders [18]. Therefore, the use of BTX-A in children require specific informed consent and protocol approved by our ethical committee.

## 9. Special Cases: Reduced Compliance

It has been shown that therapy with BTX-A is most effective in neurogenic bladders with overactive detrusor. This applies to bladders with noninhibited contractions as well as to those with reflex micturition contractions at low volumes.

Over time, compliance or wall elasticity becomes deteriorated in neurogenic bladders. This alteration can be associated to overactivity of the detrusor muscle or it may be an isolated event. It has been reported that in up to 35% of the latter cases the bladders did not respond to BTX-A. Hence, it could be questioned whether this lack of response is associated to an increase in collagen tissue deposit in the detrusor wall, especially observed in cystoscopic studies where trabeculae and/or diverticula were found.

Paradoxically, some nonresponding patients exhibit improvement in their micturition volumes as evidenced in voiding diaries, and urinary continence also improves; therefore, these patients experience substantial enhancement in their quality of life. Moreover, the continuous subjective improvement reported by patients or their families after 9 months of receiving botulinum toxin injections is surprising, although these results are not reflected in urodynamic parameters. This discrepancy could be explained by afferent changes triggered by different pathways, such as suburothelial afferent nerves [17, 23].

Some medical teams have evaluated the possibility of using botulinum toxin type B in cases of resistance to BTX-A. This toxin seemed to be more efficient and safer for those patients; however, the duration of action was shorter than that observed in patients who used BTX-A [24].

Another alternative to therapeutic resistance is to analyze the synergy between two different subtypes of botulinum toxin. Nevertheless, to be able to confirm these assumptions it is necessary to design protocols with stricter criteria of patient selection.

## 10. Repeat Injections

Because the effects of BTX-A in the detrusor muscle are reversible due to nerve regeneration, repeated injections are necessary in order to keep a sustained therapeutic effect. Grosse et al. showed that repeated injections are as effective as the first one, when using 300 U of BOTOX or 750 U of Dysport. Some patients received up to 7 repeat injections [25]. The repetition of injections every 6 to 9 months leads to a sustained improvement over time [15, 26]. However, there are no studies in the literature that indicate whether the injections should be repeated at preestablished regular intervals or when worse clinical or urodynamic results appear after an acceptable response.

It is recommended to repeat the injections no earlier than 3 months after the first injection, in order to avoid the development of neutralizing antibodies which would attempt against the efficacy of future injections. If after the first two injections there is no clinical or urodynamic response, physicians are advised against repeat injections.

## 11. Fibrosis

In the pediatric population, the increase of collagen deposit in the bladder plain muscle fibers is a common finding in patients with the physiopathological congenital condition of neurogenic bladder [27]. Histological studies have shown the appearance of an inflammatory infiltrate with lymphocyte predominance and bladder wall edema after injection/s of botulinum toxin. These findings can also be attributed to injury of endoscopic injection.

Several studies in adults have reported on the effect of BTX-A on the detrusor wall with the use of repeated injections. No significant changes with respect to the collagen deposit or fibrosis in the detrusor before and after one or several injections of the neurotoxin have been observed [28, 29]. In 40 children with neurogenic bladder, after several BTX-A intradetrusor injections, it was shown that there was no increase of fibrosis in the bladder wall, confirming the long-term safety of this treatment. In addition, with the repeat injections a reduction in fibrosis and wall trabeculation was achieved. Botulinum toxin, in blocking the release of acetylcholine, diminishes muscular contraction and the nerve growth factor. It also inhibits release of inflammatory mediators which induce fibrosis [20, 21].

## 12. Neutralizing Antibodies

The neurotoxin causes chemical paralysis of the detrusor muscle. The quantity of toxin that leaks into the general blood circulation and may potentially produce antibodies is minimum. Furthermore, most of the subjects tested already had antitoxin antibodies before receiving the toxin; moreover, antibodies have been found in normal control subjects. The existence of these antibodies is explained because some individuals with genetic predisposition had had contact with the microorganism, which was associated to the consumption of certain preserved food [30].

A relationship between antibodies in serum and the lack of response to the toxin in neurogenic bladder has been described. This lack of efficacy or partial therapeutic response has been associated with high doses of BTX-A or its administration during short periods of time, less than 2 months, when there are high levels of antitoxin antibodies. Despite these findings, the increase of serum titers is transitory and multiple injections do not stimulate the immune system to produce neutralizing antibodies. In the same way, the immunologic memory after repeated injections could not be proved. Nevertheless, it is recommended that repeat injections be administered after 6 months of one effective injection in order to avoid increase of antibody titers, anaphylactic reactions, or treatment failure [22].

## 13. Adverse Effects

The occurrence of adverse effects in the use of botulinum toxin type A accounts for less than 10% of cases. Muscular weakness or asthenia can appear between 2 weeks and 2 months after injection. The incidence of this effect lies between 2.2% and 6%. Patients with cerebral palsy who also receive botulinum toxin due to spasticity, or those patients who had received high doses of the toxin are more likely to suffer unwanted effects [31].

Another possible effect is the increase in postvoid residual urine volume for which clean intermittent catheterization is required transitorily. The most vulnerable populations are patients with nonneurogenic dysfunctions or those who are not under clean intermittent catheterization programs [32]. The combination of toxin injection into the detrusor muscle and into the bladder neck could benefit these patients.

Local effects on the bladder have also been described: hematoma at the injection site, with reported hematuria of 3.2% to 5%. Urinary tract infections vary between 6.4% and 35%. Constipation has been reported up to 10% and autonomic dysreflexia has been less frequently reported [18]. Other less probable side effects include: diplopy, dysphagia, blurred vision, and Risk of respiratory depression. Effects in distant muscles and subclinical changes in respiratory heart rate variation need for further studies on other autonomic organs.

Botulinum toxin injection is contraindicated in those cases in which the neuromuscular junction could be blocked, for example, myasthenia gravis or aminoglycoside intake. Additionally, the use of this toxin is not recommended in cases of bleeding diathesis, hemophilia, pregnancy, or obstructed urinary tract.

## 14. Facts Up To 2011-2012

The facts are as follows.

- Optimal clinical and urodynamic results in overactive detrusor (level of evidence or LoE: 3) [18].
- More knowledge about botulinum toxin therapeutic effects in cases of neurogenic bladder due to myelomeningocele (93%). Other etiologies of neurogenic bladder are infrequent, then the experience with the toxin is limited.
- Dosage: 5–12 U/kg of body weight. Maximum dose: 300 U (grade of recommendation: B) [18].
- Start of therapeutic effect: 2 weeks.
- Maximum intensity of therapeutic effect: 4–6 weeks.
- Duration of therapeutic action: 3–6 months. Maximum duration of action: 9–12 months.
- Simultaneous injection of the toxin to the external urethral sphincter is possible.
- Improvement in intestinal function and activity of the pelvic floor. Common neural pathway can be responsible for the positive effects in bladder and bowel function.
- Still not FDA-approved for lower urinary tract and disorders of the pelvic floor.

## 15. Uncertainties

The uncertainties include the following.

- Which is the minimum level of bladder compliance acceptable to be injected? The loss compliance is related with increased connective tissue.
- Can botulinum toxin be indicated in the presence of severe urologic conditions, such as hydronephrosis or renal disease?
- Empirical use in overactive bladder syndrome; without previous urodynamic studies.
- What is the recommended dilution for each injection?
- Number of injections? Injection sites?

Better stated as current ranges with need for clarification or testing other doses and numbers of sites.

- Adjuvant antimuscarinic agents?
- Lowest reported age: 3 years old (grade of recommendation: C) [18].

## 16. Conclusion

Botulinum toxin type A has become a second-line treatment for neurogenic and nonneurogenic bladder dysfunction refractory to anticholinergic drugs. Its main beneficial effects are observed in neurogenic bladders with overactivity of the detrusor muscle, with improvement in continence and urodynamic parameters. Moreover, the procedure of introducing the neurotoxin into the bladder is ambulatory and has minimal adverse effects.

Nevertheless, further clinical randomized studies with a greater number of patients, better inclusion criteria, methods injection, and longer follow-up periods are needed in order to optimize the levels of evidence and grades of recommendation for the use of this toxin.
